# Improved segmented modified Look-Locker inversion recovery T_1_ mapping sequence in mice

**DOI:** 10.1371/journal.pone.0187621

**Published:** 2017-11-09

**Authors:** Maryam Nezafat, Isabel T. Ramos, Markus Henningsson, Andrea Protti, Tamer Basha, René M. Botnar

**Affiliations:** 1 Division of Imaging Sciences & Biomedical Engineering, King’s College London, London, United Kingdom; 2 Department of Medicine, Beth Israel Deaconess Medical Center and Harvard Medical School, Boston, Massachusetts, United States of America; 3 Cairo University, Biomedical Engineering Department, Giza, Egypt; 4 Pontificia Universidad Católica de Chile, Escuela de Ingeniería, Santiago, Chile; Universitatsklinikum Wurzburg, GERMANY

## Abstract

**Object:**

To develop and evaluate a 2D modified Look-Locker (MOLLI) for high-resolution T_1_ mapping in mice using a 3T MRI scanner.

**Materials and methods:**

To allow high-resolution T_1_ mapping in mice at high heart rates a multi-shot ECG-triggered 2D MOLLI sequence was developed. In the proposed T_1_ mapping sequence the optimal number of sampling points and pause cardiac cycles following an initial adiabatic inversion pulse was investigated in a phantom. Seven native control and eight mice, 3 days post myocardial infarction (MI) after administration of gadolinium were scanned. Two experienced readers graded the visual T_1_ map quality.

**Results:**

In T_1_ phantoms, there were no significant differences (<0.4% error) between 12, 15 and 20 pause cardiac cycles (p = 0.1, 0.2 and 0.6 respectively) for 8 acquisition cardiac cycles for 600bpm in comparison to the conventional inversion recovery spin echo T_1_ mapping sequence for short T_1_’s (<600 ms). Subsequently, all in-vivo scans were performed with 8 data acquisitions and 12 pause cardiac cycles to minimize scan time. The mean native T_1_ value of myocardium in control animal was 820.5±52 ms. The post-contrast T_1_ measured 3 days after MI in scar was 264±59 ms and in healthy myocardium was 512±62 ms. The Bland-Altman analysis revealed mean difference of only -1.06% of infarct size percentage between T_1_ maps and LGE.

**Conclusions:**

A multi-shot 2D MOLLI sequence has been presented that allows reliable measurement of high spatial resolution T_1_ maps in mice for heart rates up to 600bpm.

## Introduction

The mouse model of myocardial infarction (MI) has been increasingly used to investigate functional and molecular processes including metabolism, remodeling and energetics during the healing phase post MI in order to identify biomarkers that may predict the development of heart failure [1, 2]. However, the small size of the heart and the high heart rates in mice create several challenges and impose limitations for mouse imaging using cardiac MR. Therefore, many imaging protocols routinely used in clinical cardiac MR are difficult to apply in small animals and require adaptations. The main challenges are the rapid heart rate in mice of 450–600 bpm (RR interval: 100–150 ms) and the high-spatial resolution required (~200–300 microns) for reliable (accurate infarct size, functional parameters and tissue characterization) cardiac imaging in mice. Hence, it is important to develop new preclinical protocols to satisfy these two requirements.

Quantitative cardiac T_1_ mapping of the myocardium is an emerging imaging technique and has been shown to provide valuable diagnostic information for the assessment of several myocardial pathologies including hypertrophic and dilated cardiomyopathy, myocarditis, amyloidosis and myocardial remodeling after MI [3]. Several previous studies have shown that T_1_ mapping in preclinical research can be used to detect the presence of edema in the myocardium [4, 5] and allow visualization and quantification of gadolinium labeled stem cells [6]. Several methods for measuring the T_1_ relaxation time in patients have been introduced mostly based on Look-Locker sequences with steady state free precession (SSFP) readout [7, 8] to acquire single shot images along the M_z_ relaxation curve after an initial saturation or inversion prepulse. The single shot 2D Modified Look-Locker (MOLLI) sequence is the most widely used T_1_ mapping technique in humans but not feasible in mice due to the high heart rates and requirements on spatial resolution. T_1_ mapping methods are based on the acquisition of multiple images at different inversion times in order to obtain images with different T_1_ weighting along the recovery curve. Acquisition duration to acquire a single shot image at each heart beat is almost 150 ms to 200 ms. However, there is not adequate time to acquire the single shot image at end diastole in a mouse with a heart rate interval of 100 ms to 150 ms. Therefore, segmented k-space approaches can be used to overcome this limitation. Furthermore, for high resolution imaging in small animals, which typically requires long repetition times the FLASH-based readout is more robust because it is less sensitive to field inhomogeneities as well as cardiac and respiratory motion [9, 10].

A few T_1_ mapping methods for the mouse heart have been proposed on higher magnetic field preclinical MR scanners. A single-slice inversion recovery Look-locker sequence has been proposed and has been successfully applied on 7T and 11.7T scanners [11, 12]. However, this method has several drawbacks such as low quantitative accuracy due to the fact that several echoes were recorded per heart beat to improve the imaging speed and through-slice motion between excitations can decrease the accuracy of the sequence [13]. Another method for the measurement of T_1_ of mouse myocardium is 3D IntraGate FLASH at 9.4T preclinical MRI scanner [13]. Although high SNR is achieved with this sequence, it takes too long to obtain a T_1_ map (20min) and during such a long time washout of the gadolinium can change the T_1_ measurements. Moreover, the saturation recovery Look-Locker (SRLL) technique has been proposed to acquire a single slice T_1_ map in 3min [14] and was recently extended to multi-slice T_1_ mapping (MSRLL) to allow for more coverage without additional time cost at a preclinical 7T scanner [15]. The drawback of the SRLL sequence is the relatively low signal-to-noise ratio (SNR) and the lower precision than that of the MOLLI sequence. In addition, T_1_ mapping on preclinical systems are restricted to a few dedicated research centers and thus not available to the many cardiac MR centers with clinical MR scanners.

The aim of this work was therefore to develop a MOLLI-based T_1_ mapping acquisition scheme that provides high precision and good SNR for high-resolution T_1_ mapping of the mouse heart at high heart rates and in the presence of rapidly switching gradients for wide spread use on 3T clinical MR scanners. Validation experiments were performed with a T_1_ phantom and simulated heart rates of up to 600 bpm to evaluate the accuracy of the proposed sequence. Furthermore, the feasibility of this sequence for in-vivo imaging of myocardial infarction has been investigated in mice with permanent coronary occlusion after the administration of a gadolinium based contrast agent.

## Materials and methods

### Pulse sequence scheme

**Fig 1:**
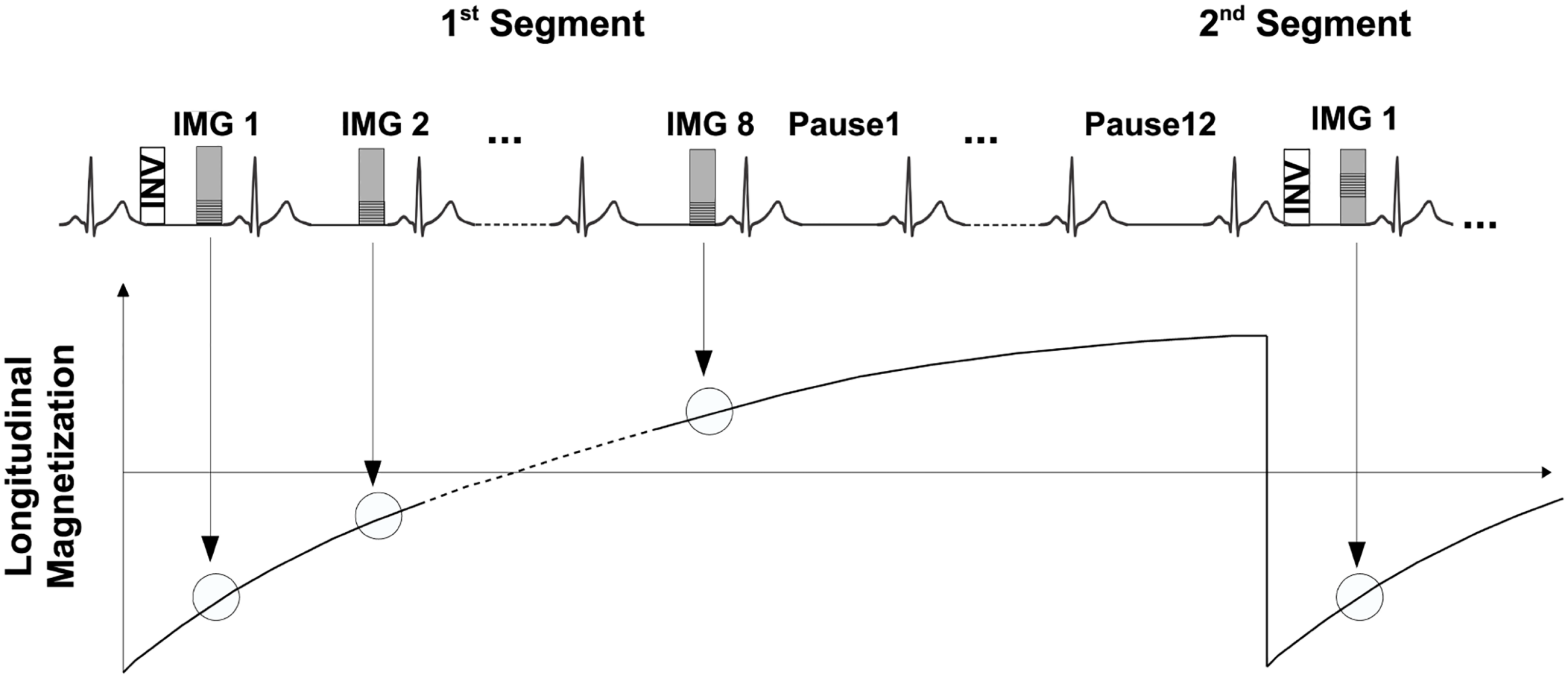
A segmented ECG triggered 2D MOLLI sequence with 8 multi-shot T_1_-weighted images and 12 RR pauses. Images were acquired in mid-diastole to minimize cardiac motion artifacts. Calculation of the T_1_ maps was performed online using a 3-parameter fit implemented in the scanners reconstruction software.

The proposed T_1_ mapping pulse sequence is shown in Fig 1 and enables myocardial T_1_ measurements in mice. It is based on the segmented ECG-triggered 2D MOLLI sequence (1 read-out per segment) with acquisition of 8 sets of T_1_-weighted images and 12 pause cardiac cycles for magnetization recovery to adapt to the high heart rates in mice. Multiple T_1_ weighted images were acquired with a multi-shot gradient echo imaging sequence. Data acquisition was performed in end-diastole to minimize cardiac motion. To minimize ECG distortions due to rapid gradient switching and RF interferences, both RF and gradients were disabled during the pause cycles. In addition, adaptive filtering of the inbuilt vector ECG electronics was utilized to additionally minimize RF and gradient interferences during data acquisition. A segmented Turbo Field gradient echo (TFE) sequence was used as readout. Optimized imaging parameters of the sequence included: FOV = 35 × 35 mm^2^, TR/TE = 7.6/ 3.1 ms, voxel size = 0.3 × 0.3 mm^2^, flip angle = 16°, slice thickness = 1.5 mm, number of profiles = 1 and scan time = 3:49 minutes. A pixel-wise 3-parameter fit reconstruction as typically used for the standard MOLLI sequence was performed using the scanners inline reconstruction software with:

$$I(t)=A-B{e^{-\frac{t}{T_{1}^{*}}}}^{}$$ (1)

where I(t) is the measured signal at the time point t after the inversion pulse, A and B are the fitting constant and T_1_* is the effective longitudinal relaxation time. The T_1_ relaxation time is then calculated using the following correction term:

$$T1=T_{1}^{*}(\frac{B}{A}-1)$$ (2)

### Phantom study

The influence of different number of pauses (12, 15 and 20 cardiac cycles) on the accuracy of the technique was investigated in a T_1_ phantom. The phantom consisted of vials containing NiCl_2_-doped agarose-gel, with varying concentration resulting in T_1_ values ranging from approximately 300 to 1200 ms. The phantom was imaged using the proposed 2D MOLLI sequence using the following parameters: TR = 2.6 ms, TE = 1.3 ms, flip angle = 16°, in-plane resolution = 2 × 2 mm^2^, FOV = 210 × 137 mm^2^. A simulated ECG signal corresponding to heart rates of 400 bpm (150 ms RR interval), 500 bpm (120 ms RR interval) and 600 bpm (100 ms RR interval) were used.

For reference, an inversion recovery 2D spin-echo sequence (IR-SE) with 16 different inversion times between 50 and 3000 (50, 100, 200, 300, 400, 500, 600, 700, 800, 900, 1000, 1250, 1500, 1750, 2000 and 3000 ms) were used. Relevant imaging parameters included TR = 10 s, TE = 11 ms, flip angle = 90°, resolution = 1.2 × 1.2 mm^2^, FOV = 300 × 131 mm^2^, slice thickness = 8 mm. All reference scans were performed using a simulated ECG with heart rate of 400 bpm.

### In-vivo study

#### Animals with myocardial infarction

All in vivo procedures were conducted in accordance with the Guidance on the Operation of the Animals and institutional guidelines. The study was approved by the UK Home Office (the application number is PPL No. 70/8482). MI was induced in eight female C57BL/6J mice (Charles River, United Kingdom) weighing between 18–24 g by permanent ligation of the left anterior descending coronary artery. Mice were anaesthetised by intraperitoneal injection of 75 mg/Kg ketamine (VetalarTMV, Vetmedica, USA) and 1 mg/Kg medetomidine hydrochloride (Domitor^®^, Orion Corporation, Finland), and 30 minutes prior to recovery, 0.15 mg/kg Buprenorphine (Vetergesic^®^, Alstoe, UK) was administered by intramuscular injection for analgesia. Mice were ventilated using a delicate small animal ventilator (Hugo Sacks Elektronic, Germany). A left thoracotomy was performed in the fourth intercostal space, the pericardium removed, and the left anterior descending coronary artery ligated permanently with an 8–0 nylon suture, at the level between 1 and 2 mm below the tip of the left atrium. Successful ligation was confirmed by regional blanching of the left ventricle, extending to the apex. After thoracotomy, subcutaneous tissue and skin were closed in separate layers and the animal weaned from the ventilator. After the surgery the mice were monitored and maintained on the heater chamber overnight.

Imaging was performed 3 days after surgery 20 to 40 minutes after i.v. administration of 0.5 mmol/kg (commonly used dose in mice to have sufficient delayed enhancement [16]) gadopentetate dimeglumine (Magnevist; Bayer Schering Pharma, Berlin, Germany). Scan parameters were the same for control and infarcted animals. After completion of MR imaging, mice were euthanized under deep anaesthesia, and hearts were excised. For 2,3,5-Triphenyltetrazolium chloride (TTC) staining, 4 animals with myocardial infarction hearts were frozen for 30 minutes and cut in 1mm slices using a specialized mouse heart slicer (Zivic Instruments, Pittsburgh, PA) that allows preparation of 1-mm-thick slices. Heart slices were then incubated with 1.5% TTC solution for 15 minutes followed by formalin fixation (4% paraformaldehyde for 1 hour at room temperature), and scanned with CanonScan LIDE70.

#### Animal imaging

Seven healthy (control) mice and eight infarcted mice were imaged in prone position with the proposed T_1_ mapping method. Animals were anesthetized with 2% isoflurane and 98% oxygen and anaesthesia was maintained throughout the entire scanning session. In vivo, scans were performed using a 3T MR scanner (Achieva, Philips Healthcare, Best, the Netherlands) equipped with a ^1^H surface coil for signal detection. The body temperature of the mice was maintained at 35 ± 1°c with warm water tubes and a temperature feedback system (SA Instruments, Stony Brook, NY) to keep the heart rate constant. The heart rates of mice with MI were 420 ± 30 bpm (cycle length 134 to 153 ms) and for control mice 500 ± 40 bpm (cycle length 115 to 130 ms). ECG tracing was derived from two metallic needles placed subcutaneously into the front paws of the mouse.

#### Cine imaging

Cine imaging was used in both infarcted and healthy mice to acquire dynamic short axis images throughout the cardiac cycle. A retrospectively ECG triggered segmented turbo field 2D gradient echo sequence was employed. 12 frames were acquired per cardiac cycle. Imaging parameters of the optimized sequence included TR/TE = 14/7.2 ms, FOV = 35 × 35 mm^2^, flip angle = 20°, in-plane resolution = 0.2 × 0.2 mm^2^ slice thickness = 1 mm and scan time = 19 seconds per slice.

#### Look-Locker (LL)

LL sequence [17] was performed 20 to 40 minutes after IV injection of 0.5 mmol/kg gadopentetate dimeglumine (Magnevist; Bayer Schering Pharma, Berlin, Germany) for MI mice to find the correct inversion time to null the signal of healthy myocardium. The 2D LL sequence consisted of an adiabatic inversion pulse which was applied immediately after the detection of the R-wave and followed by segmented gradient echo readout. Imaging parameters were TR/TE = 16/7.2 ms, flip angle = 10°, in-plane resolution = 0.5 × 0.5 mm^2^, slice thickness = 2 mm and scan time = 25 second. The time between subsequent inversion pulses was 5 heartbeats to allow for adequate recovery of the longitudinal magnetization.

#### 3D LGE imaging

The 3D late gadolinium enhancement (LGE) inversion recovery (IR) sequence comprised 8 different slices and was performed with the inversion time determined with the LL sequence in order to maximize the contrast between healthy and infarcted myocardium (240 ± 30 ms). For image acquisition a segmented gradient echo sequence was used with Cartesian k-space sampling and linear profile order. The imaging parameters were TR/TE = 6.8/2.8 ms, flip angle = 25°, in-plane resolution = 0.3 × 0.3 mm^2^, slice thickness = 1 mm and scan time = 5:22 minutes for a heart rate 450 bpm. The proposed T_1_ mapping sequence was performed at the slice position that showed the largest area of infarction.

### Analysis

All data were presented as mean ± standard deviations and P≤0.05 is considered statistically significant. Linear regression analysis was used for phantom experiments to describe the relationship between calculated T_1_ value with the proposed 2D MOLLI sequence and reference. T_1_ maps were generated on a pixel wise basis online for the proposed MOLLI sequence. Two expert readers scored the in-vivo T_1_ maps quality using a five-point scale system: 1: poor quality, 2: structured visible but markedly blurred, 3: Anatomy visible but with moderate blurring, 4: minimal blurring, 5: excellent quality. Reference T_1_ times were calculated by applying a three-parameter fit model offline.

Average T_1_ relaxation times were then calculated after manually drawing a region of the interest (ROI) in the phantom vials, remote myocardium and infarct using OsiriX software v.5.5.2. In the phantom study, the precision was calculated as the average over the standard deviation of the ROI’s drawn on each vial. The left ventricular scar size percentage was calculated as follows:

$$Infarct\;Area=[\sum_{apex}^{base}(\frac{{area}_{infarct}}{{area}_{infacrt}+{area}_{remote}})*100]/number\;of\;slices$$ (3)

Where *area*_*infarct*_ is the infarcted area and the *area*_*remote*_ is the non-infarcted part of the left ventricular. The area of the infarct and remote was measured by manually drawing a ROI around the area of interest in Osirix for LGE and T_1_ maps. ImageJ 1.46 was used to measure the infarct area of the TTC images.

## Results

### Phantom study

**Fig 2:**
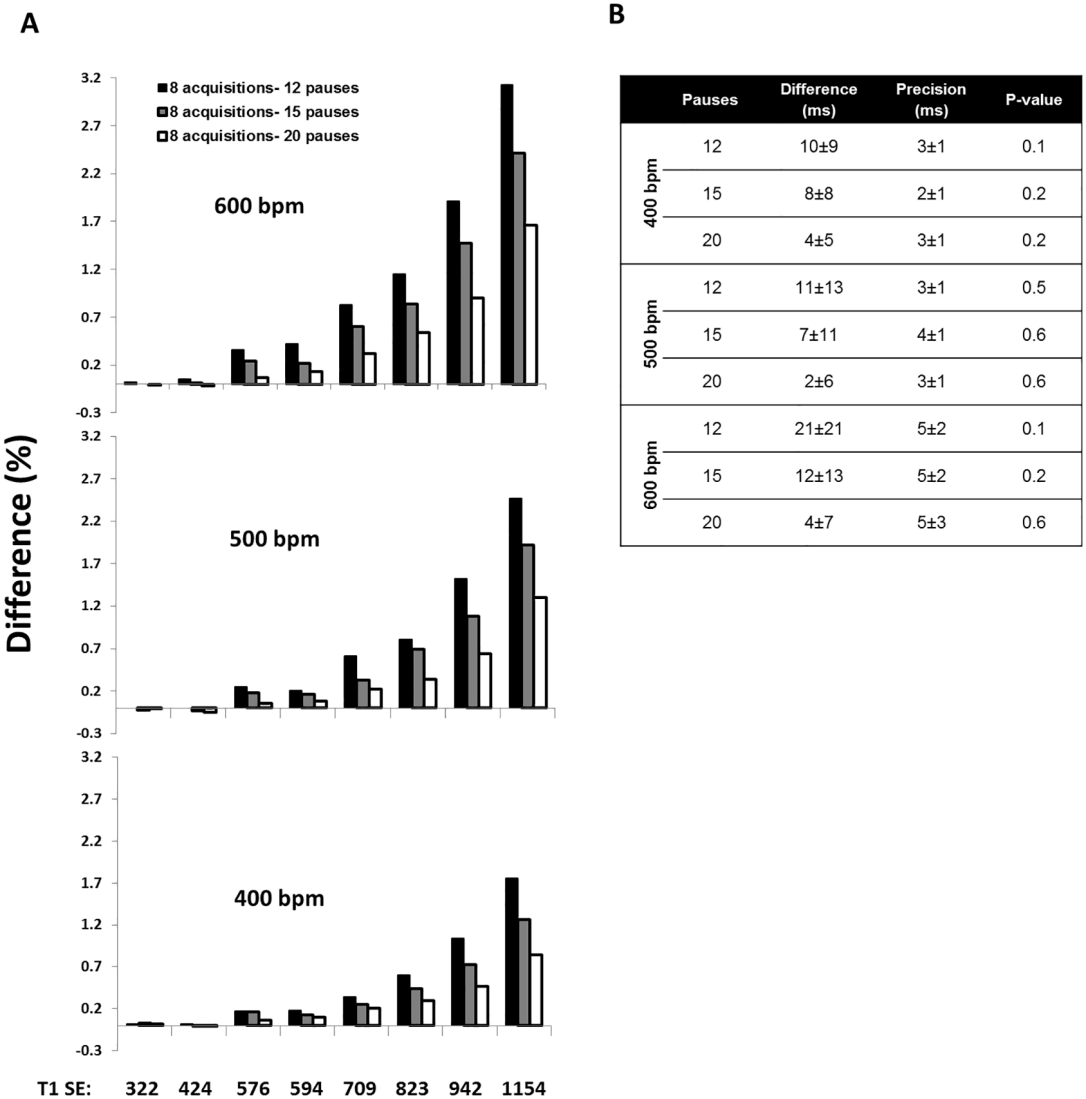
**A)** T_1_ values of vials calculated with 8 acquisitions and three different numbers of pauses cardiac cycles (12, 15 and 20) in comparison with a spin echo reference sequence (bottom) for heart beats of 400, 500 and 600 bpm. For short T_1_ (T_1_<600ms) the differences are 0–0.2%, 0–0.2% and % 0–0.4% for long T_1_ (600 ms < T_1_<1200 ms), they are 0.2–1.76%, 0.2–2.5% and 0.3–3.1% for 400 bpm, 500 bpm and 600 bpm, respectively. **B)** The relative difference between the reference and segmented MOLLI, precision and p-value for short T_1_ (<600) for heart rates of 400 bpm, 500 bpm and 600 bpm. SE: Spin Echo.

Fig 2A shows the percentage difference between T_1_ values for 400 bpm, 500 bpm and 600 bpm and the IR-SE sequence as a reference. For short T_1_ (T_1_<600ms) the differences are 0–0.2%, 0–0.2% and % 0–0.4%, for long T_1_ (600 ms < T_1_<1200 ms), they are 0.2–1.76%, 0.2–2.5% and 0.3–3.1% for 400 bpm, 500 bpm and 600 bpm, respectively (Fig 2A). No significant differences were found between 12, 15 and 20 pauses for short T_1_ values (T_1_<600ms) for all three heart beats (Fig 2B p-value).

### In-vivo study

According to the phantom results, 8 acquisitions and 12 pauses were deemed sufficient for all in-vivo experiments to minimize scan time.

#### Control mice

**Fig 3:**
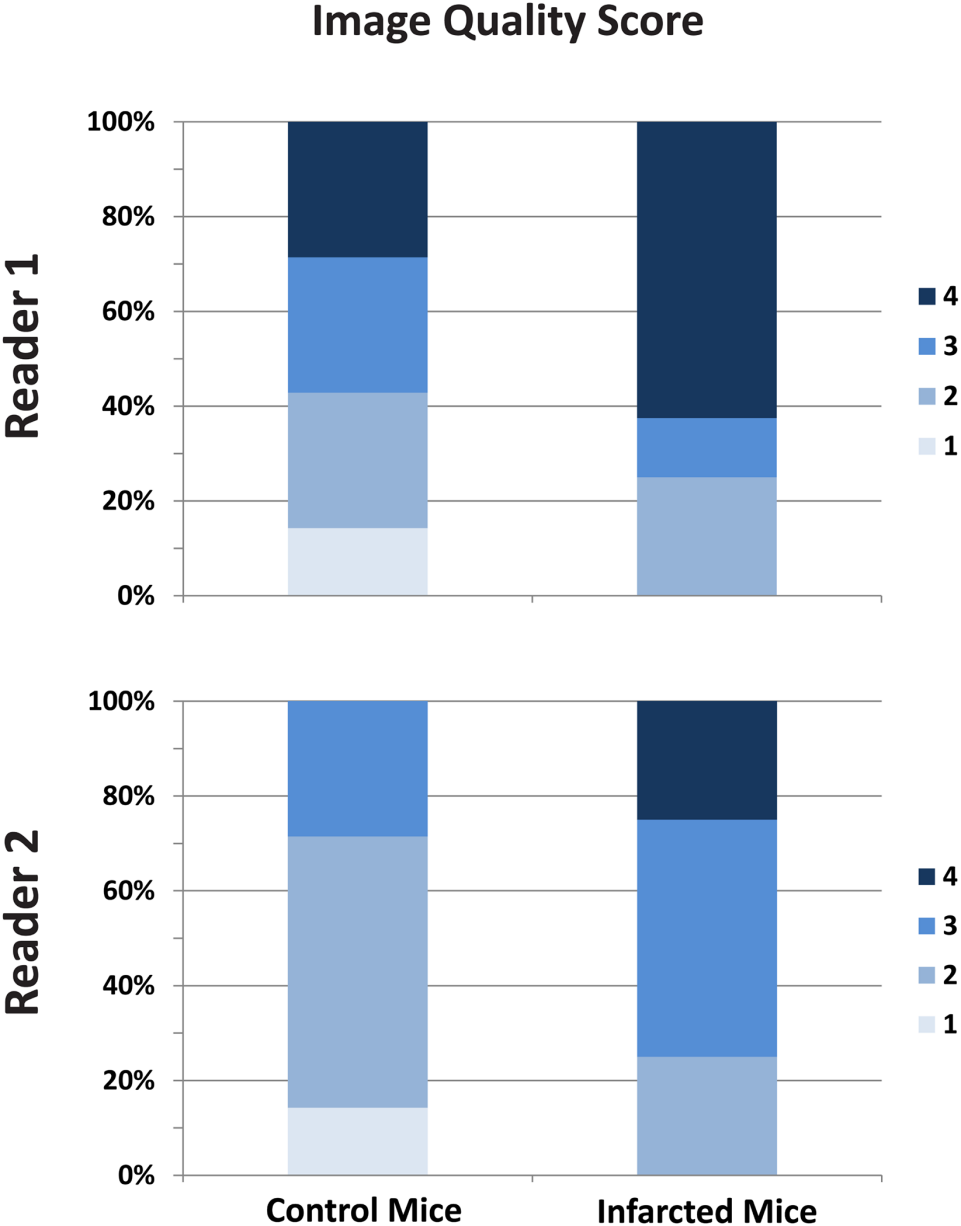
The T_1_ map quality scores of the healthy and infarcted mice. 1: poor quality, 2: structured visible but markedly blurred, 3: Anatomy visible but with moderate blurring, 4: minimal blurring, 5: excellent quality.

**Fig 4:**
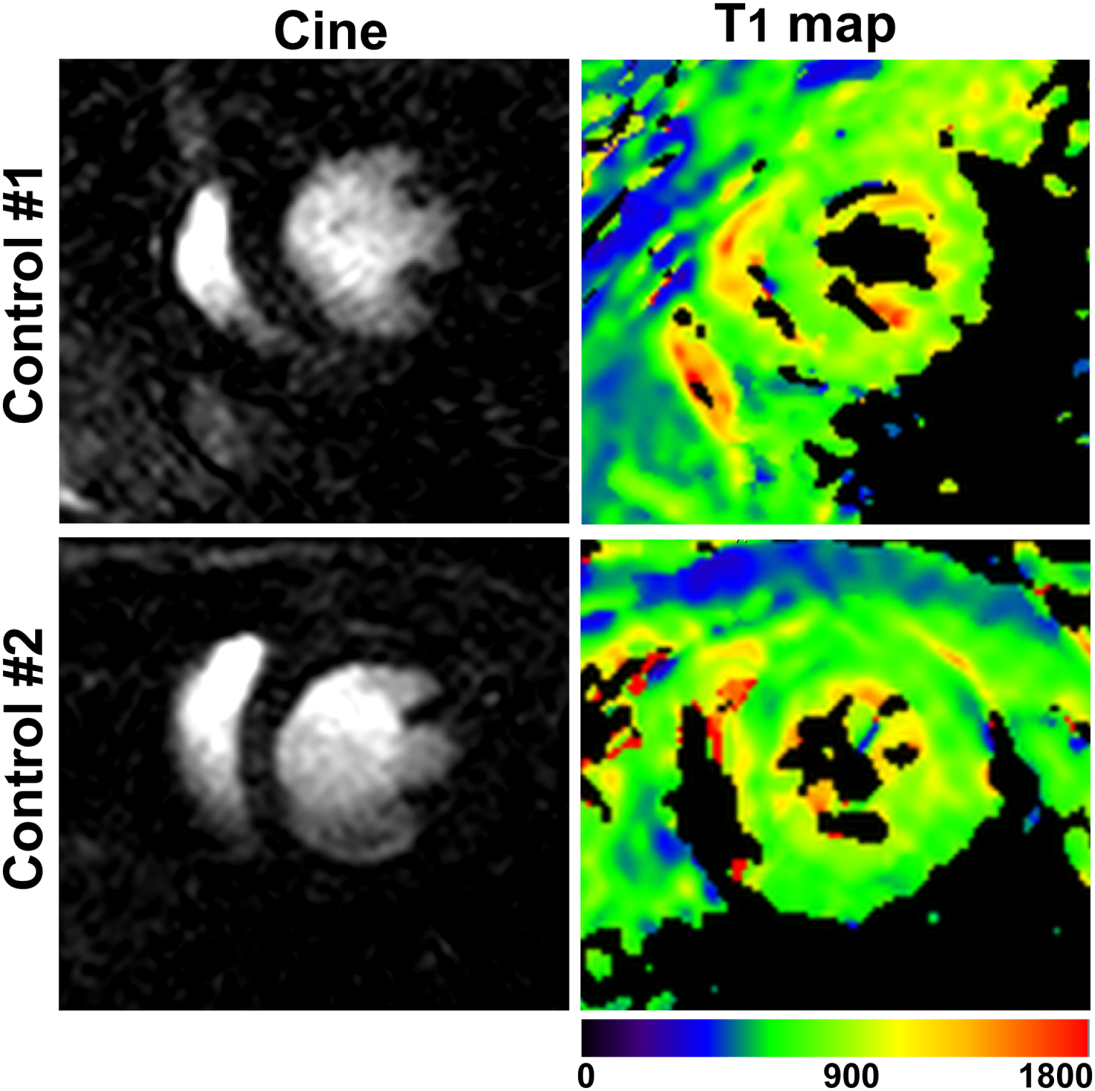
**Example of native T**_**1**_ **maps with the proposed sequence in two control animals** (right column). The cine images are shown (left column) to allow visual comparison of the morphological structures.

Cine and T_1_ map images were successfully acquired in seven control mice (S1 Fig). The T_1_ map quality score was 2.4 ± 0.9 (Fig 3). The inter-observer agreement for quality scores of the maps of infarcted mice and control mice provided by proposed sequence showed fair agreement, with an ICC of 0.56 (95% confidence interval: 0.06 to 0.84). Fig 4 shows representative cine images and T_1_ maps of two healthy control mice. The mean of the T_1_ relaxation time value in controls pre-contrast injection was 820.5 ± 52 ms.

#### Infarcted mice

**Fig 5:**
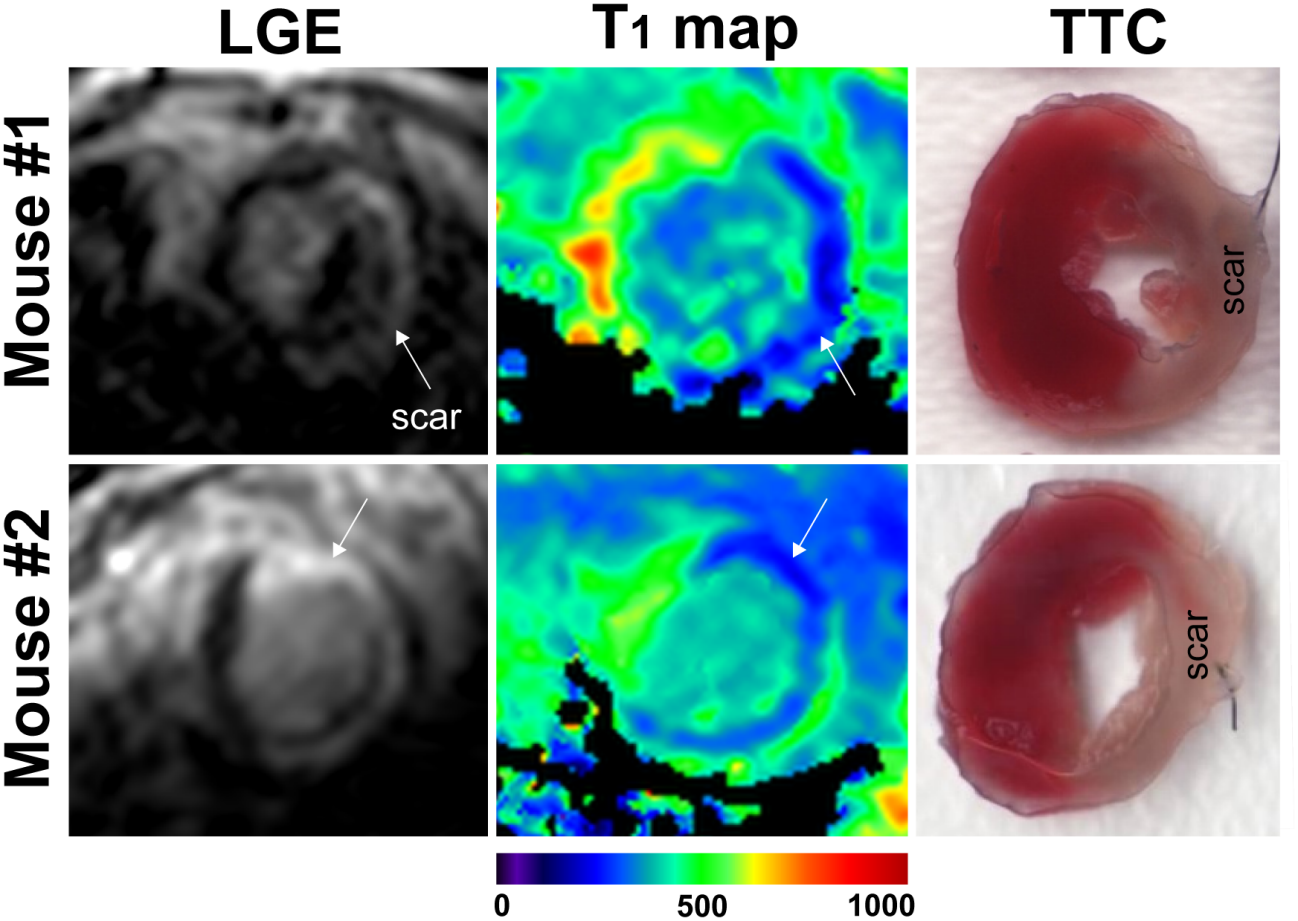
Representative post-contrast images acquired 3 days post MI with 3D LGE sequence (left column) and 2D MOLLI T_1_ maps (middle column). For visual comparison histology of the mouse heart with an infarct is being shown in the right column. The red tissue corresponds to viable myocardium and the pale tissue is scar. MI: myocardial infarction; LGE: late gadolinium enhancement, TTC: Triphenyltetrazolium chloride.

**Fig 6:**
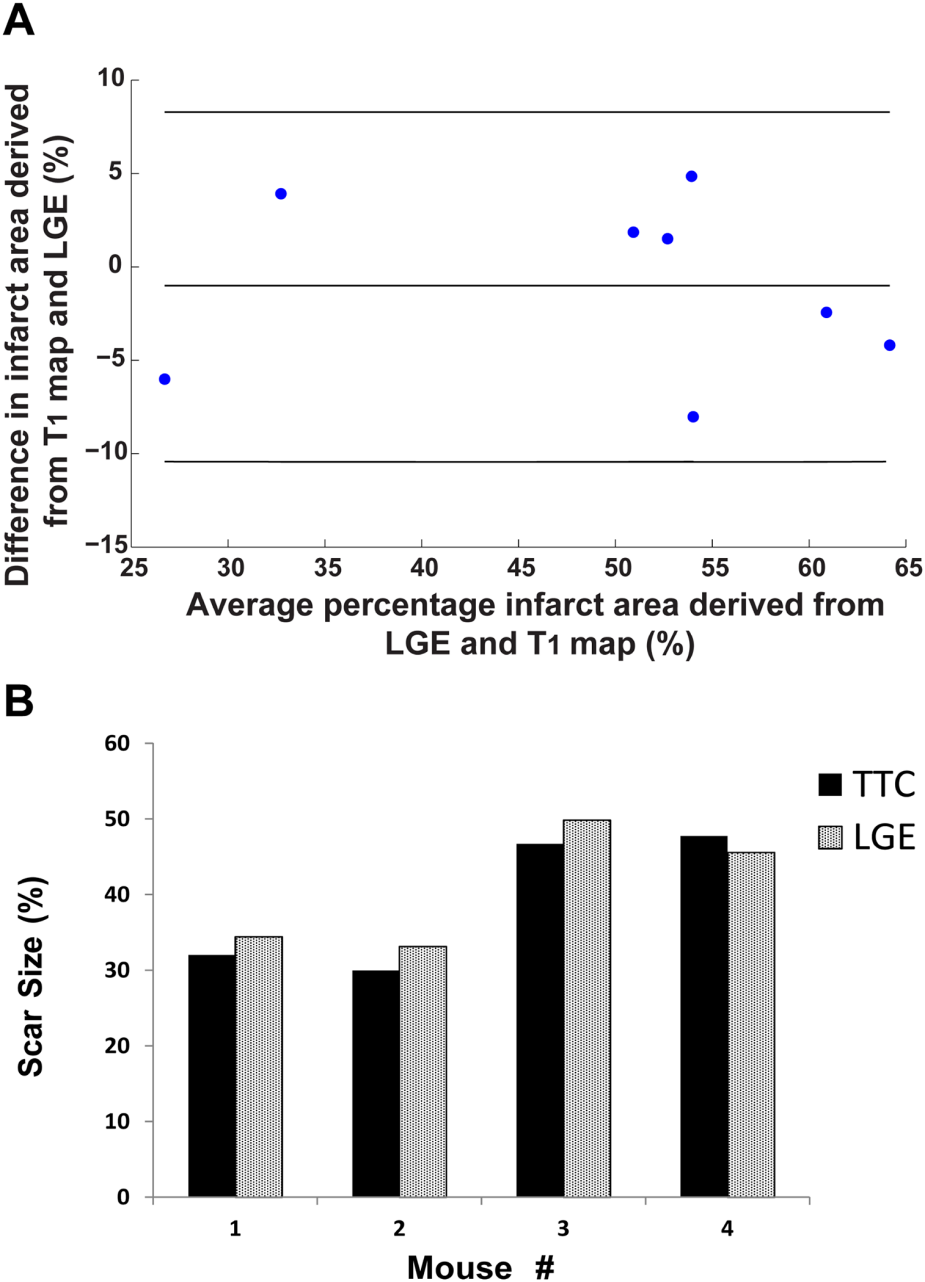
A) Bland-Altman plots of infarcted area derived from LGE images and T_1_ maps of eight infacted mice; B) Percentage infarct area for the LGE and TTC for all slices in 4 mice.

Imaging was successfully completed in all infarcted animals (S2 Fig). The T_1_ map quality score was 3.2 ± 0.8 (Fig 3). Fig 5 shows representative LGE images, T_1_ maps and histology of the heart from two infarcted mice. There was good agreement between infarct tissue (infarct: pale area, viable myocardium: red-stained area) visualized by histology and the corresponding LGE images. The measured post-contrast T_1_ values of infarct and healthy myocardium in MI mice were 264 ± 59 ms and 512 ± 62 ms, respectively. The average percentage of the infarcted area that was measured from T_1_ maps, was 48.9 ± 13% which resulted in agreement with that found by the LGE scan of 50 ± 13% (p = 0.5). The Bland-Altman analysis was performed to assess the correlation between individual measures of infarct size (Fig 6A). The mean difference between infarct size calculated from LGE and the T_1_ maps was -1.06%, with upper and lower 95% limits of agreement at -10.4% and 8.2%, respectively. Fig 6B shows the comparison of the scar size measured in T_1_ maps and the histology for 4 mice.

## Discussion

In this study, we present a segmented 2D MOLLI T_1_ mapping sequence that allows for measurement of myocardial T_1_ in mice at high heart rates and with high spatial resolution. T_1_ phantom measurements demonstrated good agreement between the proposed segmented MOLLI sequence and the reference spin echo T_1_ mapping sequence for high heart rates and T_1_ relaxation times between 300 and 1200 ms. The proposed sequence was successfully used in-vivo and provided good to excellent T_1_ mapping quality in the majority of the cases.

Our finding was that image quality was better in infarcted mice than in control mice. This is because the T_1_ values in infarcted mice were lower than in the control mice due to the injection of a contrast agent (820.5 ± 52 ms vs. 512 ± 62 ms in normal myocardium). According to our phantom study, the calculated T_1_ value with the proposed sequence is more accurate for short T_1_ (less than 600) especially for higher heart rates (600 bpm). The reason for this is that for short T_1_ values there are enough measurement points along the steep part of recovery curve due to complete recovery of the magnetization. Thus image quality and accuracy of the T_1_ maps is improved for post-contrast T_1_ mapping compared to pre-contrast T_1_ mapping. In order to use the sequence for tissues with longer T_1_, the number of pauses needs to be increased to allow the magnetization to completely recover. As can be seen form the phantom results, there is less T_1_ error for 20 pauses compared to 12 or 15 pauses, especially for longer T_1_ values.

The Small Animal Look-Locker Inversion Recovery (SALLI) for simultaneous generation of T_1_ maps and cine at clinical scanner has been proposed by Messroghli et al [18]. Although SALLI creates T_1_ maps and IR-prepared with one sequence, in a case that native T_1_ maps are needed such as myocardial edema, the data needs to acquire both pre-contrast (use for calculating native T_1_ maps) and post-contrast (use for late gadolinium enhancement). The accuracy of the cine and IR-prepared images of the SALLI sequence has not been evaluated. Further studies needs to compare the accuracy of T_1_ maps that calculated with our proposed sequence and SALLI sequence.

MOLLI is the most commonly used myocardial T_1_ mapping technique in humans [7]. Despite the underestimation of the calculated T_1_, which results from sensitivity of the sequence to heart rate, B_0_ and B_1_ inhomogeneities, imperfect Look-locker correction, imperfection of the adiabatic inversion pulse and a deflection of the relaxation curve caused by the serial read-outs pulses [19–21], MOLLI has a robust performance in terms of excellent image quality, excellent precision and reproducibility of the measurements.

The sampling interval was calculated from the average heart rate during the acquisition. However, the variation in heart rate causes inaccuracy in T_1_ estimation. In our experiments, we minimized heart rate variation by maintaining the animal’s body temperature at a constant level using water based heating system combined with a temperature feedback system to reduce this effect on T_1_ estimation.

In our study respiratory gating was not used. Previous studies have shown that respiratory motion has little impact on T_1_ mapping in mice [22]. We did not observe significant motion artefacts in images of mice under isoflurane anaesthesia, most likely due the low ratio between breathing and heart rate [23], Thus no respiratory gating was deemed necessary, which led to a significant time saving. However, in some cases there are artefacts due to the respiratory motion such as thicker septum in mouse #2 in Fig 4. We did not correct for this issue, because the image quality is good enough to show the area of the infarction. The segmented MOLLI-based sequence can be susceptible to artifacts due to heart rate variation or missed R-waves which may lead to T_1_ errors. This issue could be addressed using ‘inversion time gating’, where data is discarded if the expected TI is very different from actual TI. However, this leads to prolonged scanning time, like any gating method and thus was not used in this study. Instead, we chose to maintain the body temperature at 35C using a water based heating system which resulted in a stable heart rate in all animals. We did not compare our sequence to the heart rate independent saturation recovery Look-Locker (SASHA) technique [14] which could be an alternative approach. As the dynamic range and SNR of the SASHA technique is smaller than that of MOLLI it has lower precision and is less reproducible while the accuracy is improved [24].

A number of potential limitations must be addressed. We investigated a small number of controls, myocardial infarcted mice and histology; further studies will be needed on a larger scale. The calculated infarct size from the proposed sequence has been compared to the LGE rather than TTC, due to the fact that two in-vivo tissue properties are the same. In TTC, the method to prepare the tissue, may cause the scar area to shrink or deform that may make error in calculating the scar size. Secondly, by using the clinical scanner the image quality is lower compare to the preclinical scanner. Another limitation is that phantom study has been done using a 32-element cardiac coil, due to the reason that having the small clinically validated T_1_ phantom is challenging. Therefore, the optimized parameters in the phantom are different than the one that we used in mice.

## Conclusions

In conclusion, an ECG triggered, segmented preclinical 2D MOLLI based sequence was developed for cardiac T_1_ mapping in mice and was validated in mice with and without myocardial infarction.

## Supporting information

S1 Fig Cine, native T_1_ maps and T_1_ errors for 7 control animals. T_1_ error for each segment was calculated as the mean over the standard deviation of the T_1_ values of the segment.
(TIF)

S2 Fig Cine, post-contrast T_1_ maps, TTC and T_1_ errors for infarcted animals. (TIF)
