# Supplementary figures and images for: Improved segmented modified Look-Locker inversion recovery T1 mapping sequence in mice
Source: PLoS One. 2017 Nov 9;12(11):e0187621. doi: 10.1371/journal.pone.0187621 (PMC5679534; doi:10.1371/journal.pone.0187621)

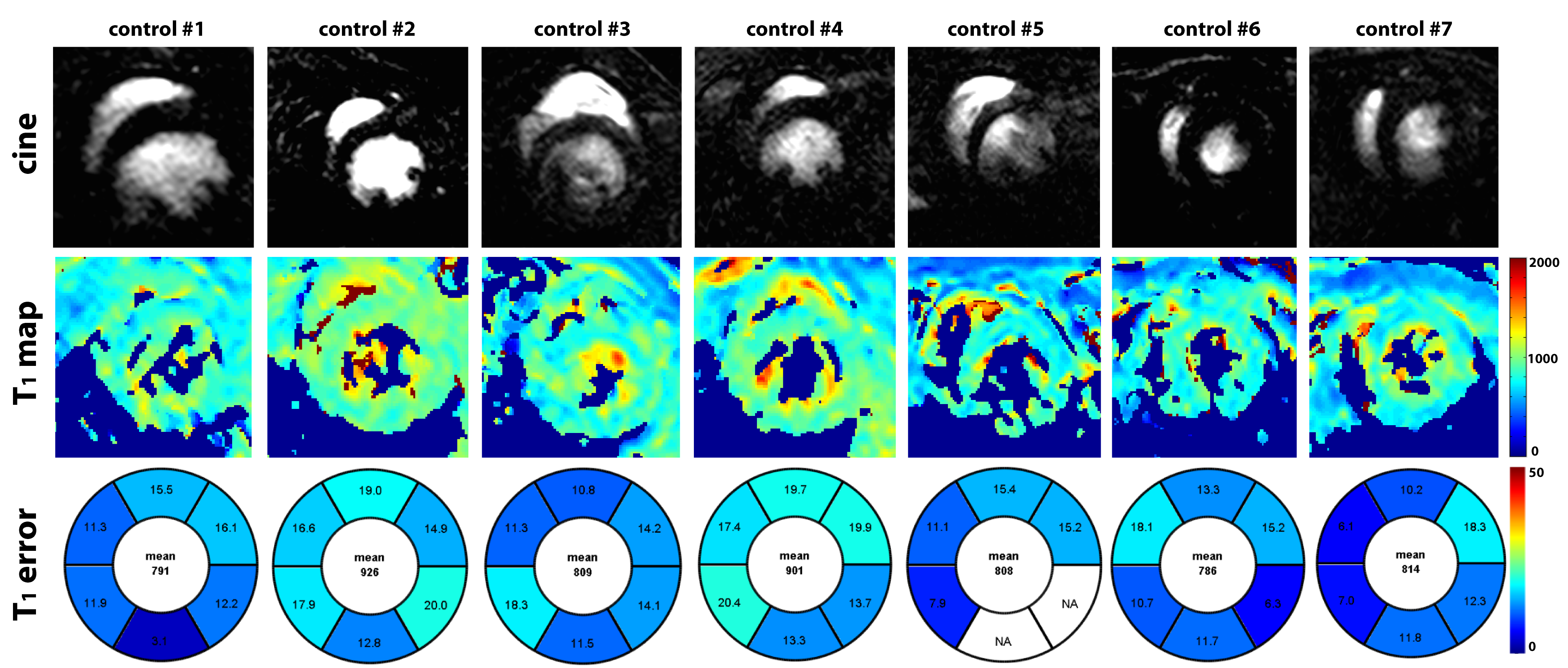

Supplement: S1 Fig — T1 error for each segment was calculated as the mean over the standard deviation of the T1 values of the segment. (TIF) [file pone.0187621.s001.tif]

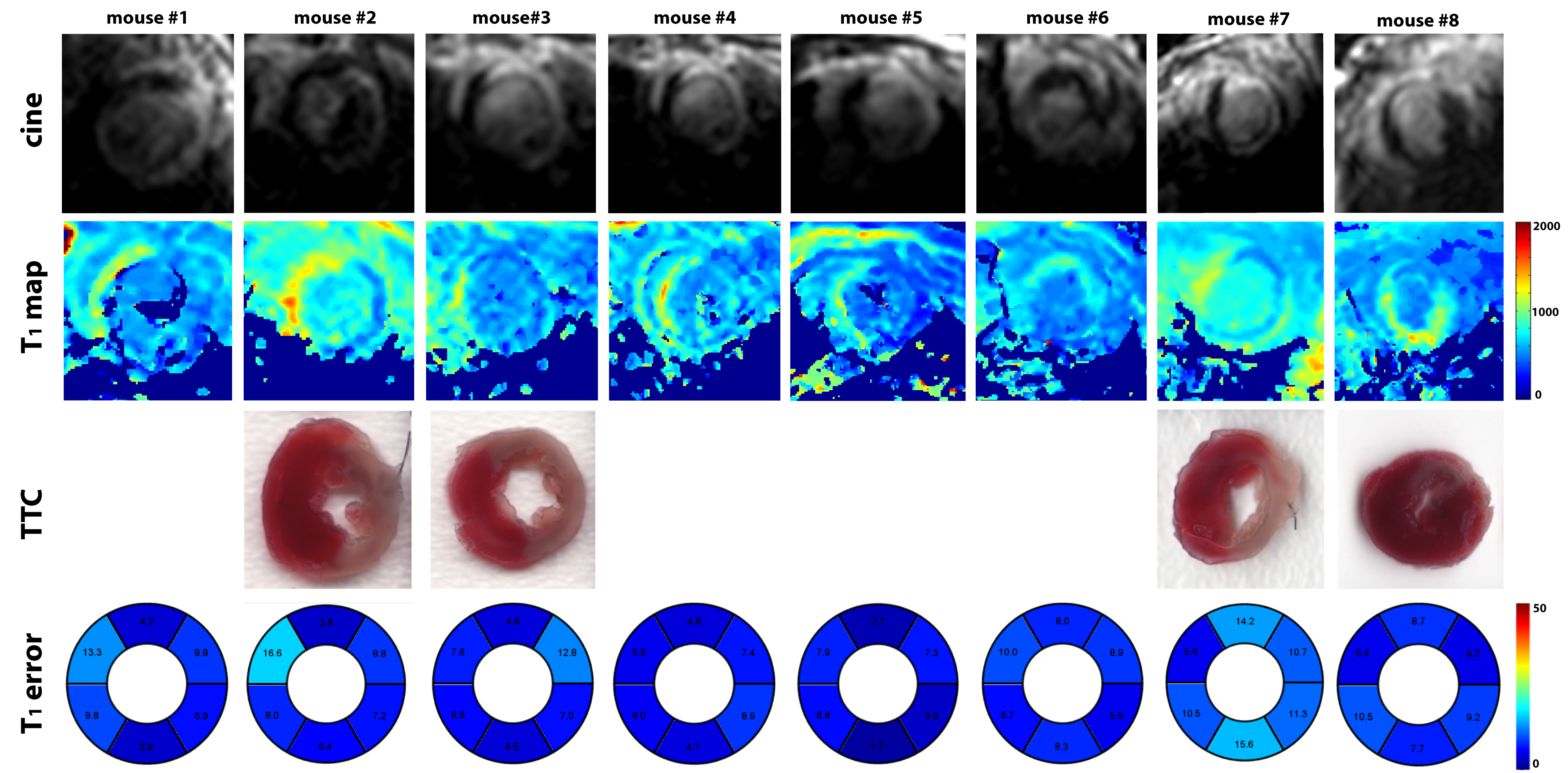

Supplement: S2 Fig — (TIF) [file pone.0187621.s002.tif]
